# Blood biomarkers of neuronal injury in paediatric cerebral malaria and severe malarial anaemia

**DOI:** 10.1093/braincomms/fcad323

**Published:** 2023-11-27

**Authors:** Dibyadyuti Datta, Adnan Gopinadhan, Alejandro Soto, Paul Bangirana, Robert O Opoka, Andrea L Conroy, Andrew J Saykin, Keisuke Kawata, Chandy C John

**Affiliations:** Ryan White Center for Pediatric Infectious Disease and Global Health, Indiana University School of Medicine, Indianapolis, IN 46202, USA; Ryan White Center for Pediatric Infectious Disease and Global Health, Indiana University School of Medicine, Indianapolis, IN 46202, USA; Department of Microbiology and Immunology, Indiana University School of Medicine, Indianapolis, IN 46202, USA; Ryan White Center for Pediatric Infectious Disease and Global Health, Indiana University School of Medicine, Indianapolis, IN 46202, USA; Department of Psychiatry, Makerere University College of Health Sciences, P.O. Box 7072, Kampala, Uganda; Global Health Uganda, P.O. Box 33842, Kampala, Uganda; Global Health Uganda, P.O. Box 33842, Kampala, Uganda; Aga Khan University Medical College, P.O. Box 30270, Nairobi, Kenya; Ryan White Center for Pediatric Infectious Disease and Global Health, Indiana University School of Medicine, Indianapolis, IN 46202, USA; Indiana Alzheimer’s Disease Research Center and Department of Radiology and Imaging Sciences, Indiana University School of Medicine, Indianapolis, IN 46202, USA; Department of Kinesiology, Indiana University School of Public Health-Bloomington, Bloomington, IN 47405, USA; Program in Neuroscience, The College of Arts and Sciences, Indiana University, Bloomington, IN 47405, USA; Ryan White Center for Pediatric Infectious Disease and Global Health, Indiana University School of Medicine, Indianapolis, IN 46202, USA

**Keywords:** severe malaria, CNS injury biomarkers, child development, neurologic deficits, cognitive impairment

## Abstract

Persistent neurodisability is a known complication in paediatric survivors of cerebral malaria and severe malarial anaemia. Tau, ubiquitin C-terminal hydrolase-L1, neurofilament-light chain, and glial fibrillary acidic protein have proven utility as biomarkers that predict adverse neurologic outcomes in adult and paediatric disorders. In paediatric severe malaria, elevated tau is associated with mortality and neurocognitive complications. We aimed to investigate whether a multi-analyte panel including ubiquitin C-terminal hydrolase-L1, neurofilament-light chain, and glial fibrillary acidic protein can serve as biomarkers of brain injury associated with mortality and neurodisability in cerebral malaria and severe malarial anaemia. In a prospective cohort study of Ugandan children, 18 months to 12 years of age with cerebral malaria (*n* = 182), severe malarial anaemia (*n* = 158), and asymptomatic community children (*n* = 118), we measured admission blood levels of ubiquitin C-terminal hydrolase-L1, neurofilament-light chain, and glial fibrillary acidic protein. We investigated differences in biomarker levels, associations with mortality, blood–brain barrier integrity, neurodeficits and cognitive *Z*-scores in survivors up to 24-month follow-up. Admission ubiquitin C-terminal hydrolase-L1 levels were elevated >95th percentile of community children in 71 and 51%, and neurofilament-light chain levels were elevated >95th percentile of community children in 40 and 37% of children with cerebral malaria and severe malarial anaemia, respectively. Glial fibrillary acidic protein was not elevated in disease groups compared with controls. In cerebral malaria, elevated neurofilament-light chain was observed in 16 children who died in hospital compared with 166 survivors (*P* = 0.01); elevations in ubiquitin C-terminal hydrolase-L1 levels were associated with degree of blood–brain barrier disruption (*P* = 0.01); and the % predictive value for neurodeficits over follow-up (discharge, 6-, 12-, and 24 months) increased for ubiquitin C-terminal hydrolase-L1 (60, 67, 72, and 83), but not neurofilament-light chain (65, 68, 60, and 67). In cerebral malaria, elevated ubiquitin C-terminal hydrolase-L1 was associated with worse memory scores in children <5 years at malaria episode who crossed to over 5 years old during follow-up cognitive testing [*β* −1.13 (95% confidence interval −2.05, −0.21), *P* = 0.02], and elevated neurofilament-light chain was associated with worse attention in children ≥5 years at malaria episode and cognitive testing [*β* −1.08 (95% confidence interval −2.05, −1.05), *P* = 0.03]. In severe malarial anaemia, elevated ubiquitin C-terminal hydrolase-L1 was associated with worse attention in children <5 years at malaria episode and cognitive testing [*β* −0.42 (95% confidence interval −0.76, −0.07), *P* = 0.02]. Ubiquitin C-terminal hydrolase-L1 and neurofilament-light chain levels are elevated in paediatric cerebral malaria and severe malarial anaemia. In cerebral malaria, elevated neurofilament-light chain is associated with mortality whereas elevated ubiquitin C-terminal hydrolase-L1 is associated with blood–brain barrier dysfunction and neurodeficits over follow-up. In cerebral malaria, both markers are associated with worse cognition, while in severe malarial anaemia, only ubiquitin C-terminal hydrolase-L1 is associated with worse cognition.

## Teaser

Datta *et al*. report elevated ubiquitin C-terminal hydrolase-L1 (UCH-L1) and neurofilament-light chain (NF-L) in paediatric severe malaria. In cerebral malaria, NF-L is associated with mortality; UCH-L1 is associated with blood–brain barrier dysfunction and neurologic deficits over follow-up. Both markers are associated with worse cognition in cerebral malaria; UCH-L1 is associated with worse cognition in severe malarial anaemia.

## Graphical Abstract

**Graphical Abstract:**
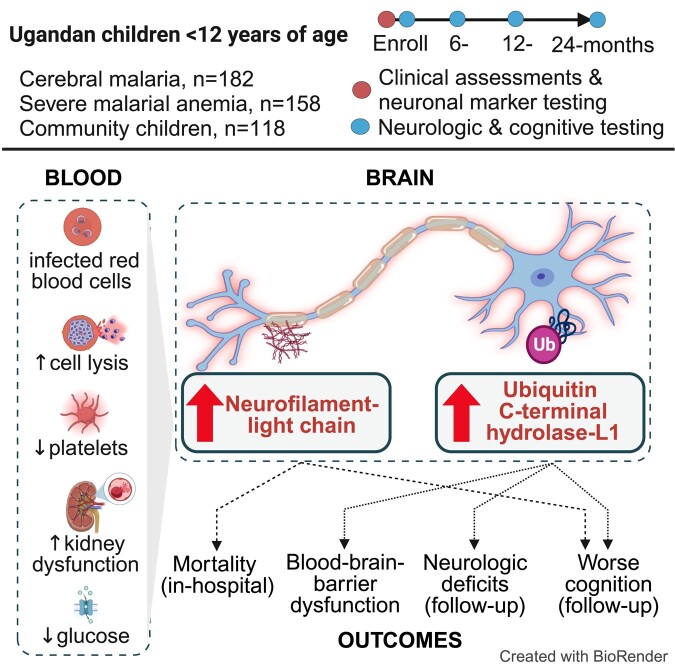

## Introduction

Severe falciparum malaria is a major driver of childhood mortality and persisting cognitive impairment in Africa. Severe complications leading to adverse outcomes in this multi-system disease include coma, severe anaemia, metabolic dysfunction including lactic acidosis and hypoglycaemia, coagulopathy, and acute kidney injury (AKI).^1,2^ Central to the pathogenesis of severe malaria is the sequestration of parasites to the endothelium, widespread inflammation, systemic accumulation of toxic by-products from parasite invasion into red blood cells, subsequent haemolysis and cellular lysis, and endothelial barrier dysfunction.^3^ Without crossing the blood–brain barrier (BBB), malaria parasites can significantly impact brain function resulting in cognitive impairment, whether a child presents with clinical signs of neurologic complications, as seen in cerebral malaria, or without, as in severe malarial anaemia.^4,5^

Biomarkers of systemic inflammation, endothelial activation, and organ dysfunction have been identified as risk factors for mortality and cognitive impairment in severe malaria.^6-8^ Less is known about the role of brain injury biomarkers that reflect CNS damage and can differentiate between neuronal, axonal, or astroglial injury,^9,10^ although these biomarkers have been associated with adverse neurologic outcomes in paediatric disorders such as traumatic brain injury and neonatal encephalopathy.^11,12^ In severe malaria, elevated CSF and plasma levels of axonal injury marker tau have proven utility in predicting systemic complications, mortality, and cognitive impairment, though associations with worse cognition scores were observed only in cerebral malaria and not severe malarial anaemia, limiting its usefulness as a standalone biomarker in severe malaria.^13,14^ A multi-analyte panel of injury biomarkers serving different functional roles in the CNS may mitigate the limitation of a single biomarker for predicting adverse outcomes. Further, as with tau, using ultrasensitive single-molecule detection assays for quantification of low abundance CNS proteins in blood circulation can circumvent the need for invasive lumbar punctures for CSF biomarker detection in paediatric severe malaria.^15^

Beyond tau, biomarkers that have proven utility as markers of acute CNS injury include ubiquitin C-terminal hydrolase-L1 (UCH-L1), neurofilament-light chain (NF-L), and glial fibrillary acidic protein (GFAP). UCH-L1, the most abundant protein in the CNS, is a 25-kDa protein expressed by neurons and involved in tagging proteins for ubiquitination or degradation in response to cellular damage.^16^ NF-L is a 68-kDA microstructural protein that provides a stabilizing framework for neuronal axons to support nerve conduction and contributes towards the regeneration of damaged axons.^17^ GFAP (50 kDa) is a component of cytoskeletal filaments of astrocytes that are upregulated during astrocyte activation in response to neurotrauma.^10,18^ These biomarkers are in different stages of clinical development for traumatic brain injury,^19^ have been associated with adverse neurologic outcomes in stroke,^20,21^ and are useful in predicting cognitive impairment in adults.^22-24^ Recently, UCH-L1 and GFAP were approved by the U.S. FDA as point-of-care plasma markers to rule out the need for CT scans after mild traumatic brain injury in adults.^25^ There are, however, few studies of these markers as diagnostic or prognostic tools in paediatric traumatic brain injury^26^ and none in paediatric severe malaria. Children at risk of CNS injury, particularly in resource-constrained malaria-endemic countries with limited access to advanced brain imaging technologies, may benefit from access to point-of-care brain biomarker tests to identify those at risk of future cognitive impairment. A critical first step is to determine if these biomarkers are useful predictors of complications associated with CNS injury in these at-risk paediatric populations.

To evaluate whether plasma levels of UCH-L1, NF-L, or GFAP are altered in severe malaria and associated with adverse outcomes, we measured these biomarkers in a cohort of children admitted with cerebral malaria or severe malarial anaemia, compared with asymptomatic community children, using an ultrasensitive single-molecule detection assay for measurement. We then correlated biomarker levels to BBB impairment and mortality during admission, neurologic deficits at discharge and follow-up, and cognitive outcome *Z*-scores over a 24-month follow-up. We hypothesized that plasma UCH-L1, NF-L, and GFAP levels would be elevated in children with cerebral malaria or severe malarial anaemia compared with asymptomatic community children and that the elevated levels of these biomarkers would be associated with mortality, neurologic deficits, and worse cognitive *Z*-scores long-term in survivors of severe malaria.

## Materials and methods

### Participants

The study was performed at Mulago National Referral Hospital in Kampala, Uganda from 2008 to 2015. Children were eligible if they were between 18 months and 12 years of age with cerebral malaria or severe malarial anaemia. A reference group of community children of the same age range and with no active illness were recruited from the extended family or household compounds of children with severe malaria. Cerebral malaria was defined as *Plasmodium falciparum* on blood smear, coma (Blantyre Coma Score <3 or Glasgow Coma Score <8), and no evidence of other known causes of coma (meningitis, prolonged postictal state, and hypoglycaemia reversible by glucose infusion). Severe malarial anaemia was defined as *P. falciparum* on blood smear and haemoglobin ≤ 5 g/dL. Children were managed according to the Ugandan Ministry of Health treatment guidelines at the time of the study. Additional sociodemographic information is as published.^5,13^

### Standard protocol approvals and patient consent

Written informed consent was obtained from the parents/guardians of study participants. Ethical approval was granted by the Institutional Review Boards at Makerere University School of Medicine (2008-033) and the University of Minnesota (0808M27022). The study was also approved by the Uganda National Council for Science and Technology (HS432). The study was conducted following the Strengthening the Reporting of Observational Studies in Epidemiology (STROBE) reporting guidelines for observational studies.

### Clinical and laboratory assessments

The study was conducted with samples collected at hospital admission for cerebral malaria and severe malarial anaemia and at enrolment for community children. Samples were processed and ethylenediaminetetraacetic acid (EDTA) plasma was stored at −80°C using standardized laboratory procedures, until testing. A complete blood count to enumerate haemoglobin and platelets was performed using a Beckman Coulter ACT 5 diff haematology analyzer (Beckman Coulter Eurocenter, SA). Biochemistries were performed on cryopreserved plasma samples for glucose, lactate, sodium, lactate dehydrogenase (LDH), creatinine, and blood urea nitrogen (BUN) using Roche Cobas Integra 400 plus Chemistry analyzer by the Advanced Research & Diagnostic Laboratory at the University of Minnesota. Creatinine was used to define the degree of AKI as previously described.^13^ HIV testing was performed according to the Uganda National HIV testing algorithm after obtaining consent from the parent or guardian. As part of routine testing in cerebral malaria, a lumbar puncture to collect CSF was conducted to rule out other known causes of coma in children who did not have a contraindication to a lumbar puncture and whose parents did not decline consent. Albumin was measured in CSF and plasma using a Sigma-Aldrich Bromocresol Purple Albumin assay (Advanced Research & Diagnostic Laboratory, University of Minnesota). BBB integrity was assessed by calculating the CSF to plasma albumin index categorized by degree of disruption.^27^ An index value < 9 was considered consistent with an intact barrier. Values between 9 and 14 were interpreted as slight impairment, 14 and 30 as moderate impairment, and 30 and 100 as severe impairment, with moderate and severe impairment combined in this analysis.

### Neurologic and cognitive assessments

Testing for gross neurologic deficits (composite score calculated from scores for motor deficits, ataxia, movement, speech, and visual disorders) in children with cerebral malaria, and for cognitive outcomes in children with cerebral malaria or severe malarial anaemia were performed at discharge (neurologic testing) or 1-week after discharge (cognitive testing), and at 6-, 12-, and 24-month follow-up. Neurologic testing was not done for children with severe malarial anaemia or community children. Community children underwent cognitive testing at enrolment and follow-up. Cognitive testing for children younger than 5 years included Mullen Scales of Early Learning for overall cognition, Early Childhood Vigilance Test for attention, and Color Object Association Test for associative memory, while cognitive tests for children 5 years or older included Kaufman Assessment Battery for Children (K-ABC, 2nd edition) with summary mental processing index for overall cognitive ability, the Test of Variables of Attention with D prime measure for attention, and the KABC-2 subtest for sequential processing subtest for working memory. Different cognitive testing batteries were used for children younger than 5 years and those 5 years or older; therefore, results are presented for (i) children younger than 5 years at malaria episode and follow-up malaria testing, (ii) children younger than 5 years at malaria episode who crossed to 5 years or older at follow-up testing, and (iii) children 5 years or older at time of malaria episode and follow-up testing. Age-adjusted *Z*-scores were created using scores of the Ugandan community children included in the study, and the age-range-specific cognitive assessments have been validated and used in previously published studies with Ugandan children.^28^

### Biomarker assessments

Plasma UCH-L1, NF-L, and GFAP levels were measured on samples with sufficient volume for testing between August and September 2018 using the single-molecule array (Simoa) HD-1 analyzer (Quanterix, MA, USA), with a lower limit of detection at 1.74, 0.104, and 0.221 pg/mL for UCH-L1, NF-L, and GFAP, respectively.^15^ Measurement of plasma total tau, included in this study, to show the correlation between biomarkers, was similarly conducted using the HD-1 analyzer.^13^ Samples with values for all three biomarkers were included in this study. Samples were tested blinded to participant details and 10% of samples were run in duplicate, and for these, mean biomarker values were used for analysis. Elevated UCH-L1, NF-L, and GFAP levels were established using levels >95th percentile of community children for each marker as a reference range.

### Statistical analysis

Analyses were done using Stata SE v17.0 (StataCorp., TX, USA). Differences in continuous measures were evaluated using Wilcoxon rank-sum tests or Kruskal–Wallis tests, where appropriate. Differences in categorical variables were measured using Pearson’s *χ*^2^ test. The *a priori* level of significance was 0.05 with two-tailed hypothesis tests. Spearman’s rank analysis was used to evaluate the correlation between CNS markers. The degree of BBB disruption was analysed using Cuzick's rank-based non-parametric test of trend (NP-trend).^29^ Logistic or linear regression, adjusting for age and sex, was used to evaluate associations of CNS marker levels with dichotomous or continuous outcomes, respectively. Continuous variables not normally distributed were log_10_-transformed for inclusion in regression models. To evaluate the discriminatory ability of CNS markers to predict neurologic deficits over time, non-parametric receiver operating characteristic (ROC) curve analysis was used and the area under the curve (AUC) was compared. Cognition was calculated as continuous *Z*-scores for three independent outcomes (one primary and two secondary) and analysed in a linear mixed effect (LME)^14^ model adjusted for variability in scores over time and using age-adjusted *Z*-scores from community children as the baseline to standardize cognitive scores for children with severe malaria. Evaluations for each cognitive outcome were not adjusted for multiple comparisons because the LMEs model incorporated scores from all time-points as a single outcome. Correction for multiple comparisons of biomarkers levels at four time-points without and with neurologic deficits, and of analysis of association with clinical laboratory factors was conducted using the Benjamini–Hochberg procedure.

## Results

### Baseline socio-demographic and clinical characteristics

Of the 718 children enrolled in the parent study, CNS markers were assessed in 340 children with severe malaria (182 with cerebral malaria, 158 with severe malarial anaemia) and 118 community children with sufficient plasma for testing. Children included were representative of the full cohort and baseline characteristics of children assessed for these biomarkers including a participant flow diagram, are as published.^5,13^ Children with severe malarial anaemia were younger than children with cerebral malaria and community children, and the percentage of females with cerebral malaria, severe malarial anaemia, or community children was 41, 36, and 53, respectively (Table 1). A small number of children enrolled in the study [3 of 183 children with cerebral malaria (1.8%), 5 of 158 children with severe malarial anaemia (3.2%), and 2 of 118 community children (1.7%)] were positive for HIV, and no significant differences were observed between the groups (Table 1). Clinical laboratory markers relevant to the pathophysiology of severe malaria including indicators of metabolic dysfunction (glucose, lactate, and sodium), haemolysis, and cellular dysfunction (LDH and haemoglobin levels, and platelet count) kidney dysfunction (AKI and BUN) were compared across groups (Table 1). Most clinical laboratory markers varied between children with the two forms of severe malaria compared with controls and between children with cerebral malaria or severe malarial anaemia. Of the 182 children with cerebral malaria, 16 died (8.8% mortality). None of the 158 children with severe malarial anaemia died (0% mortality).

**Table 1:** Baseline sociodemographic and clinical laboratory markers

	Cerebral malaria (*n* = 182)	Severe malarial anaemia (*n* = 158)	Community children (*n* = 118)	*P* value
Demographic characteristics				
Age, years	3.5 (2.6, 4.9)	2.9 (2.1, 4.6)	3.6 (2.6, 4.6)	**0.005** ^a^
Sex, *n* (% female)	74 (40.66)	59 (36.42)	65 (52.85)	**0.04** ^b^
HIV infected, *n* (%)	3 (1.8)	5 (3.2)	2 (1.7)	**0.62**
Clinical laboratory markers				
Glucose, mmol/L	6.8 (5.2, 9.5)	6.4 (4.8, 8.1)	N/A	0.08
Lactate, mmol/L	3.8 (2.0, 6.2)	5.2 (3.1, 8.0)	N/A	**<0.001** ^c^
Sodium, mmol/L	133 (128, 136)	134 (132, 136)	137 (135, 139)	**<0.001** ^b,c^
BUN, mg/dL	17 (11, 24)	12 (9, 19)	7 (5.1, 8.3)	**<0.001** ^b,c^
Acute kidney injury	65 (37.6)	29 (18.9)	5 (4.4)	**<0.001** ^b,c^
LDH, U/L	824 (640, 1071)	760 (609, 958)	261 (231, 304)	**<0.001** ^b^
Haemoglobin, g/dL	6.5 (5.3, 8.3)	3.9 (3.3, 4.5)	11.8 (11, 12.7)	**<0.001** ^b,c^
Platelet count, ×10	61 (34, 104)	158 (96, 243)	391(299, 452)	**<0.001** ^b,c^

Data presented as median (IQR) or *n* (%) unless otherwise stated. *P* values derived from Kruskal–Wallis’s test for continuous non-normally distributed measures and *χ*^2^ test for categorical measures. Significant *P* values indicated in bold. Data available and presented for surviving children with testing done. N/A, data not available. ^a^Children with severe malarial anaemia differ from children with cerebral malaria and community children. ^b^Children with cerebral malaria and severe malarial anaemia differ from community children. ^c^Children with cerebral malaria differ from children with severe malarial anaemia.

### Circulating blood levels of and correlation between CNS injury markers

**Figure 1:**
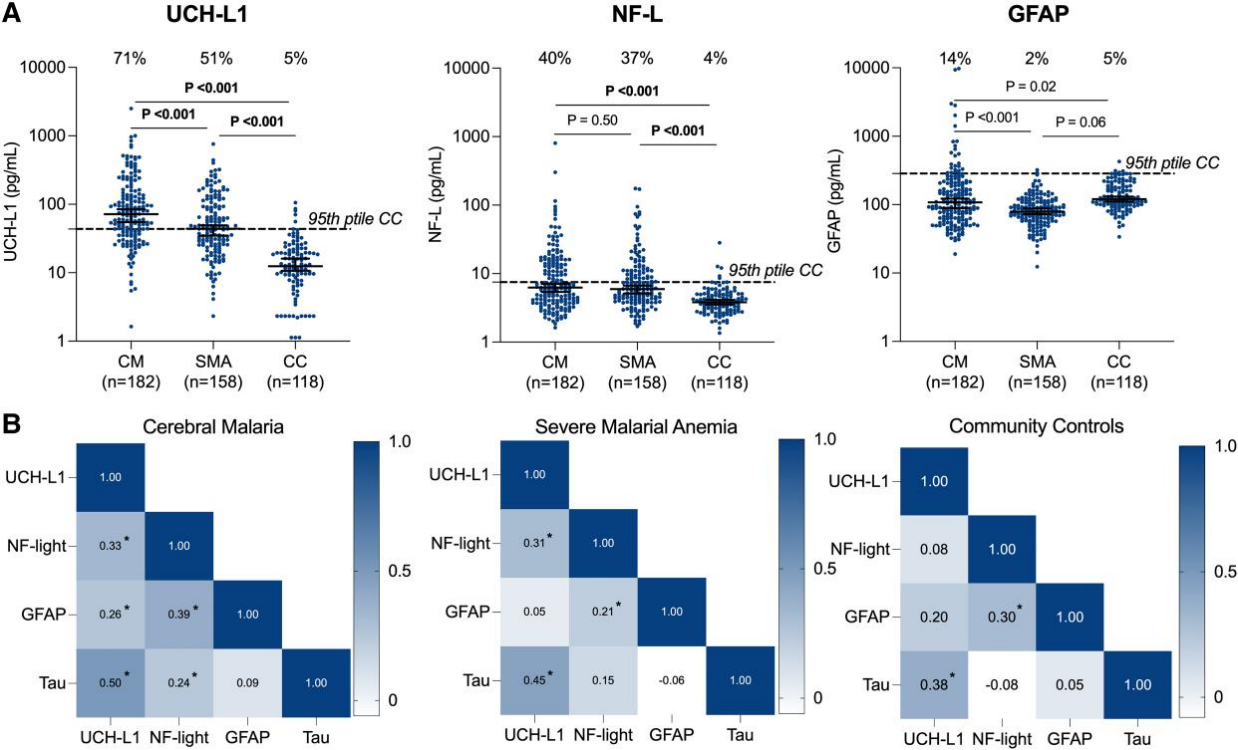
**Levels of and correlation between CNS biomarkers.** (**A**) Admission plasma biomarker concentration by study group. Dots represent biomarker concentrations (pg/mL) in individual children and solid lines show median concentrations and interquartile range. *P* values reflect two-sample Wilcoxon rank-sum test. Significant *P* values are shown in bold. The 95th percentile biomarker levels in community controls are shown with a dashed line, and the number over each bar represents the percentage in the group with biomarker levels elevated above the 95th percentile in community children. (**B**) Correlation between biomarkers and plasma tau (previously tested). Numbers within in boxes depict Spearman’s correlation coefficients. *P < 0.01. CM, cerebral malaria; SMA, severe malarial anaemia; CC, community children; UCH-L1, ubiquitin C-terminal hydrolase-L1; NF-L, neurofilament-light chain; GFAP, glial fibrillary acidic protein.

Based on the 95th percentile of community children as the cut-off for plasma UCH-L1 (43.5 pg/mL), NF-L (7.5 pg/mL), and GFAP (284.5 pg/mL), UCH-L1 levels were elevated in 71% (*n* = 130) and 51% (*n* = 81) of children with cerebral malaria and severe malarial anaemia, respectively (all *P* < 0.001), and plasma NF-L levels were elevated in 40% (*n* = 73) and 37% (*n* = 58) of children with cerebral malaria and severe malarial anaemia, respectively (all *P* < 0.001) (Fig. 1A). Median GFAP levels in children with cerebral malaria or severe malarial anaemia were not elevated compared with community children (Fig. 1A). Consequently, GFAP was excluded from further analysis of the association with BBB impairment, and in-hospital and post-discharge outcomes.

Given that age is reported as a confounder for NF-L and GFAP levels and sex for UCH-L1 levels,^16,30^ we investigated biomarker levels in children <5 versus ≥5 years of age and in female versus male children for each study group. UCH-L1 levels were significantly elevated in children <5 years of age with cerebral malaria or severe malarial anaemia and in community children compared with children ≥5 years of age (all *P* ≤ 0.05; Supplementary Table 1A). NF-L levels were elevated in children <5 years of age with cerebral malaria (*P* = 0.02) and community children (*P* < 0.001) but not in children with severe malarial anaemia (*P* = 0.10) relative to their older counterparts (Supplementary Table 1A). GFAP levels were elevated in children <5 years of age compared with older children in cerebral malaria (*P* = 0.02) and community children (*P* = 0.004) but not severe malarial anaemia (*P* = 0.31; Supplementary Table 1A). There were no significant differences in biomarker levels by sex in any group (Supplementary Table 1B).

To determine if these CNS markers can serve as complementary analytes in severe malaria, we investigated correlations between these markers and previously published Simoa tau levels.^13^ All markers correlated with each other in cerebral malaria (all *P* < 0.001) except GFAP and tau (*P* = 0.22; Fig. 1B). In severe malarial anaemia, UCH-L1 correlated with both NF-L and tau, and NF-L correlated with GFAP (all *P* ≤ 0.009). Among community children, correlations were observed between UCH-L1 and tau and between NF-L and GFAP (both *P* < 0.001; Fig. 1B).

### UCH-L1 and NF-L levels, mortality, and BBB disruption in cerebral malaria

In 16 children with cerebral malaria who died, UCH-L1 levels were not significantly elevated [median pg/mL interquartile range (IQR) 93.5 (53.6, 140.1)] compared with levels in 166 survivors [69.05 (39.08, 136.87), *P* = 0.25]. Conversely, NF-L levels were significantly higher in the children who died [median pg/mL (IQR) 11.12 (6.44, 18.88)] compared with survivors [5.93 (3.71, 13.57), *P* = 0.01].

**Figure 2:**
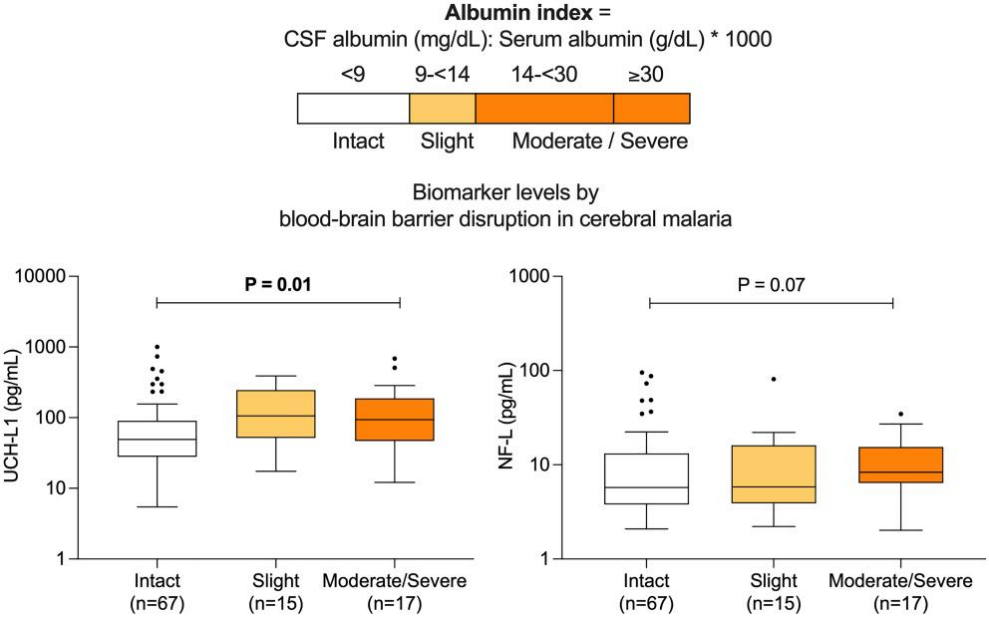
**UCH-L1, NF-L, and BBB disruption in cerebral malaria.** Albumin index used to categorize disruption as intact, slight, moderate/severe. Box and whiskers plots represent medians and interquartile ranges for UCH-L1 and NF-L concentrations, respectively, for each disruption category. Black dot represents outlier concentrations in a single child in each disruption category. *P* Values derived from Cusick’s rank-based non-parametric test of trend and shown in bold when significant. CM, cerebral malaria; UCH-L1, ubiquitin C-terminal hydrolase-L1; NF-L, neurofilament-light chain.

As BBB disruption is one mechanism implicated in the pathogenesis of cerebral malaria,^3^ we investigated whether the degree of BBB disruption was related to elevations in CNS marker levels in cerebral malaria. Only UCH-L1 levels were associated with the degree of BBB disruption (NP-trend = 0.01; Fig. 2). Specifically, median UCH-L1 levels in 15 children with slight BBB disruption [106.2 pg/mL (IQR 51.7, 246.5)] and 17 children with moderate to severe BBB disruption [93.9 pg/mL (IQR 49.5, 158.5)] were elevated compared with children with an intact barrier [median 49.2 pg/mL (IQR 28.1, 90.5)] (all *P* ≤ 0.02). NF-L levels did not show a significant increase with the degree of BBB disruption (NP-trend = 0.07; Fig. 2). BBB disruption could not be determined in severe malarial anaemia as CSF was not collected in this group.

### Associations with neurologic deficits and cognitive *Z*-scores over follow-up

**Figure 3:**
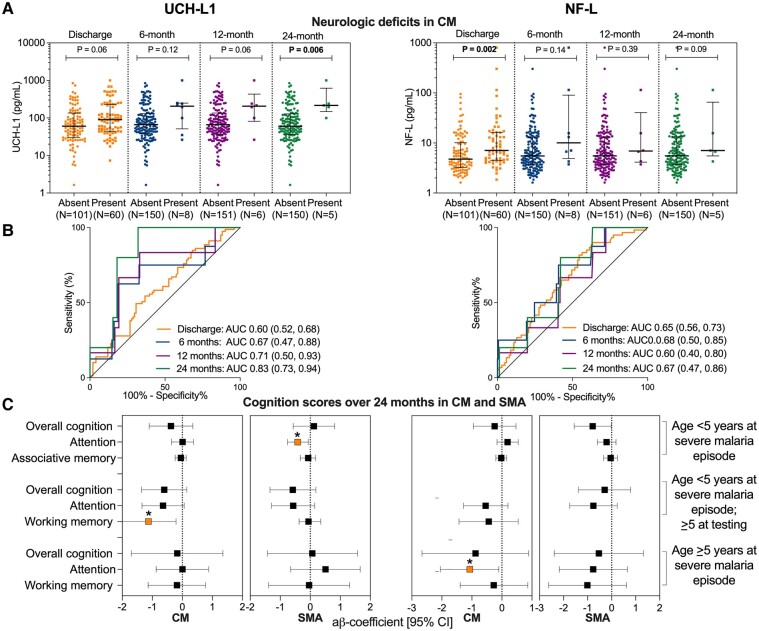
**Associations with neurologic deficits in cerebral malaria and cognitive impairment in cerebral malaria and severe malarial anaemia.** (**A**) Admission UCH-L1 and NF-L levels in children without or with neurologic deficits over follow-up in cerebral malaria. Each coloured dots represents admission UCH-L1 and NF-L concentrations in a single child, without or with neurologic deficits at discharge, 6-, 12-, and 24 months shown in yellow, blue, purple, and green, respectively. Black lines show median biomarker concentration and interquartile range. *P* values reflect a two-sample Wilcoxon rank-sum test. Significant *P* values after correction for multiple comparisons using the Benjamini–Hochberg procedure indicated in bold. (**B**) Predictive value of admission biomarkers levels with neurologic deficits over follow-up in cerebral malaria. ROC curves indicate the AUC at discharge, 6-, 12-, and 24 months shown in yellow, blue, purple, and green, respectively. (**C**) Admission biomarker levels associated with cognitive outcomes in children aged <5 years and ≥5 years, with cerebral malaria or severe malarial anaemia. Forest plot presents the adjusted *β* coefficients and 95% CIs [a*β*-coefficients (95% CI)] for biomarkers and longitudinal cognitive outcomes. The estimates were obtained using linear mixed-effects modelling adjusted for age and sex. Plot points in orange with *P < 0.01. CM, cerebral malaria; SMA, severe malarial anemia; UCH-L1, ubiquitin C-terminal hydrolase-L1; NF-L, neurofilament-light chain.

In cerebral malaria, at discharge, 6-, or 12-month follow-up, UCH-L1 levels were not different in children without or with neurologic deficits (*P* = 0.06). However, in five children with persisting neurologic deficits at 24 months, admission UCH-L1 was significantly elevated compared with children without neurologic deficits (*N* = 150, *P* = 0.006; Fig. 3A). Conversely, admission NF-L levels were elevated in children with neurologic deficits at discharge (*P* = 0.002) but not at any follow-up time-point (*P* = 0.09; Fig. 3A). These differences remained significant after correction for comparisons at four time-points using the Benjamini–Hochberg method. Complementary to these findings, the predictive value of UCH-L1 for neurologic deficits increased steadily over time (AUCs of 0.60, 0.67, 0.72, and 0.83 at discharge, 6-, 12-, and 24 months, respectively; Fig. 3B). The predictive value of NF-L did not show significant increases over time (AUCs of 0.65, 0.68, 0.60, and 0.67 at discharge, 6-, 12-, and 24 months, respectively; Fig. 3B).

After adjusting for age and sex, admission UCH-L1 levels were associated with worse z-scores for working memory over 24-month follow-up in 65 children who were <5 years of age at cerebral malaria episode but ≥5 years of age at follow-up testing {*β* −1.13 [95% confidence interval (CI) −2.05, −0.21], *P* = 0.02}. Further, UCH-L1 was associated with worse attention *Z*-scores over 24-month follow-up in 125 children with severe malarial anaemia who were <5 years of age at severe malarial anaemia episode and follow-up testing [*β* −0.42 (95% CI −0.76, −0.07), *P* = 0.02; Fig. 3C; Supplementary Table 2]. NF-L was associated with worse attention *Z*-scores over 24-month follow-up in children with cerebral malaria who were ≥5 years during severe malaria episodes and testing [*β* −1.08 (95% CI −2.05, −1.05), *P* = 0.03; Fig. 3C; Supplementary Table 2].

### Associations with known clinical risk factors in severe malaria

**Figure 4:**
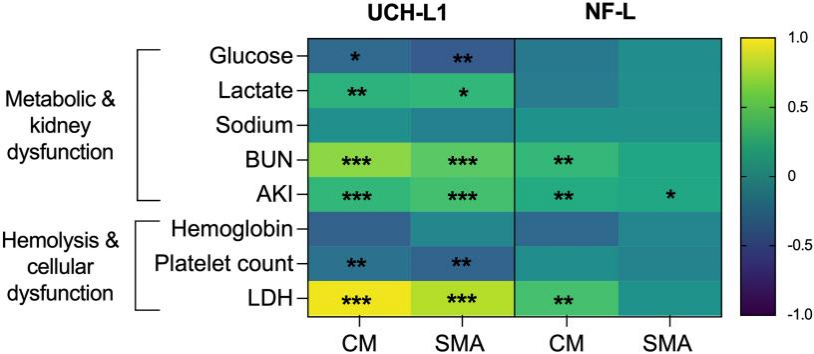
**Relationship with known clinical laboratory markers in cerebral malaria and severe malarial anemia**. Heatmap depicts β-coefficients from linear regressions adjusted for age and sex and corrected for eight laboratory factors within each marker of interest for each malaria group using the Benjamini–Hochberg procedure. *P ≤ 0.05, **P ≤ 0.01, ***P ≤ 0.001. All continuous variables were log_10_-transformed. CM, cerebral malaria; SMA, severe malarial anaemia; UCH-L1, ubiquitin C-terminal hydrolase-L1; NF-L, neurofilament-light chain; BUN, blood urea nitrogen; AKI, acute kidney injury; LDH, lactate dehydrogenase.

We explored associations of elevated UCH-L1 and NF-L levels with known clinical laboratory risk factors in severe malaria including glucose, lactate, sodium, BUN, LDH, and haemoglobin levels, platelet count, and AKI (Fig. 4; Supplementary Table 3). Elevated UCH-L1 levels were associated with several risk factors in cerebral malaria and severe malarial anaemia including low glucose, elevated lactate, platelet count, LDH and BUN, and AKI (all *P* ≤ 0.02). NF-L levels were associated with fewer risk factors primarily in cerebral malaria, namely, elevated LDH and BUN, and AKI. In severe malarial anaemia, AKI was the only risk factor associated with NF-L (Fig. 4; Supplementary Table 3). The significant associations did not change after correction for multiple comparisons.

## Discussion

In this prospective cohort study using Simoa technology for the detection of low abundance proteins, we showed that blood levels of neuronal markers UCH-L1 and NF-L, but not astrocyte marker GFAP, were elevated at hospital admission in children with cerebral malaria, who present in a coma, and in children with severe malarial anaemia, who have no clinical signs of neurologic disease. Elevations in UCH-L1 were more pronounced across disease groups and showed stronger associations with BBB dysfunction, neurologic deficits at 24 months, and worse memory scores over 24-month follow-up in younger children with cerebral malaria. In addition, elevated UCH-L1 was associated with worse attention *Z*-scores in children <5 years of age with severe malarial anaemia. Elevated NF-L was seen in children hospitalized with cerebral malaria who died and in children with neurologic deficits at discharge but not follow-up, and associated with worse attention *Z*-scores in children ≥5 years of age with cerebral malaria. The findings of this study, in conjunction with earlier findings of axonal injury in cerebral malaria and severe malarial anaemia,^13^ confirm that neuronal damage occurs in clinically diverse manifestations of paediatric severe malaria and support the hypothesis that CNS injury markers elevated above basal levels, may serve as concurrent predictors of in-hospital mortality and future cognitive impairment in children with cerebral malaria or severe malarial anaemia.

Complementary to the findings of elevated axonal microtubule marker tau,^13^ we found elevations in levels of myelinated axonal marker NF-L in children who died from cerebral malaria. In addition, concurrent elevations in UCH-L1 and NF-L levels in cerebral malaria and severe malarial anaemia compared with controls and positive correlations between these markers and tau^13^ support the validity of our findings and show that severe malaria can impact neuronal enzymatic activity as well as axonal microstructural integrities. This is further reinforced by the positive correlation between UCH-L1 and tau in controls, indicating that neuronal biomarkers jointly reflect the health of neurons regardless of disease state. Indeed, the deubiquitinating and protein degradation role of UCH-L1, the most abundant (up to 5%) among CNS proteins,^16^ has been shown to complement the axon microtubule stabilization function of tau as inhibition of UCH-L1 can decrease the microtubule-binding ability of tau and promote the accumulation of abnormal aggregates of hyperphosphorylated tau,^31^ a critical mediator implicated in cognitive impairment in neurodegenerative disorders.^32^

A novel finding of this study is that elevated admission plasma UCH-L1 levels predicted neurologic deficits at 24-month follow-up and were associated with worse associative memory scores over 24-month follow-up in children <5 years with cerebral malaria. In addition, UCH-L1 was the only CNS marker to show an association with worse attention scores in severe malarial anaemia. We noted previously that although cognitive impairment has been observed in severe malarial anaemia,^5^ elevated tau was associated with worse cognition scores in cerebral malaria alone and not in severe malarial anaemia, suggesting mechanisms other than tau-mediated axonal damage are implicated in persisting cognitive impairment in severe malarial anaemia.^13^ This study provides evidence that UCH-L1-mediated brain injury may reflect a pathway leading to impaired cognitive functioning in young children with cerebral malaria or severe malarial anaemia, although our data warrants further investigation in a larger cohort with more children per age group. In addition, elevated NF-L was associated with worse attention scores over follow-up in children with cerebral malaria ≥5 years of age at the time of severe malaria episodes. In adults with traumatic brain injury, NF-L has been found to increase with age.^33^ But in this study, all biomarkers were higher in children <5 years of age compared with those ≥5 years in disease and control groups, which likely reflects age-related structural changes rather than differences in the extent of injury. Additional studies are needed to elucidate the age-related contributions of CNS markers in paediatric brain injury and associated cognitive impairment. Although GFAP was not elevated in severe malaria compared with controls and therefore excluded from additional analysis in this study, it has been implicated as a marker of impaired cognition alongside UCH-L1 in traumatic brain injury.^22^ The high GFAP levels observed in our control populations compared with other paediatric studies may be attributed to extracerebral production as a source of GFAP in our population, or to the varying degree to which GFAP signal is detectable in the different assays used.^9,34^ As few studies have concurrently examined the role of a panel of brain injury biomarkers on persisting cognitive impairment in paediatric disorders, our findings make a strong case for further exploration of multi-analyte panels of CNS biomarkers associated with cognitive impairment including in paediatric severe malaria.

Detectable plasma levels of UCH-L1, NF-L, and GFAP in disease-free community children suggest ongoing blood circulation at basal levels in the absence of brain injury and BBB disruption, possibly from extracerebral sources, including oocytes and spermatogonia for UCH-L1, peripheral nerves for NF-L, and osteocytes, Leydig cells, and chondrocytes for GFAP.^9^ However, a more likely explanation is physiological clearance by the glymphatic system wherein fluids and small molecules are shuttled across the CNS via aquaporin-4 water channels on glial cells.^9,35^ Furthermore, the lack of association of NF-L with a degree of BBB disruption in cerebral malaria despite being significantly elevated over levels in controls suggests that BBB dysfunction is not the only mechanism underlying brain injury in severe malaria. Indeed, the validity of NF-L as a neuronal marker whose levels in blood circulation are not influenced by BBB permeability has been observed in a mouse model of brain injury.^36^ Nevertheless, elevations in the highly abundant CNS protein UCH-L1 trended positively with the degree of BBB disruption as determined by increased albumin in the CSF, which was observed in 33% of the children with cerebral malaria in this cohort. BBB dysfunction is a key component in the pathogenesis of cerebral malaria^3,37^ and a hallmark of lasting damage and neurodegeneration after traumatic brain injury.^38^ The findings of this study reflect the need for further investigations on the different mechanisms by which falciparum malaria triggers brain injury using comprehensive *in vitro* 3D models of the neurovascular unit, experimental animal models, and functional brain imaging studies in humans.

Our exploratory analysis of the relationships between CNS markers (UCH-L1 and NF-L) and clinical laboratory indicators of severe complications revealed important insights into the clinicopathogenesis of severe malaria. For example, elevated levels of UCH-L1 and NF-L were associated with hypoglycaemia (glucose <2.2 mmol/L), cellular injury (low platelet count, elevated LDH), and kidney dysfunction (AKI, elevated BUN). These complications are known to exacerbate the primary insult in traumatic brain injury^39-41^ and may increase the risk of cognitive impairment after brain injury in survivors of severe malaria. Impaired kidney function may result in the inability to eliminate biomarkers from blood circulation.^30^ Hypoglycaemia is a life-threatening complication of severe malaria that is reversed by the administration of glucose in hospitalized children to prevent mortality. However, in CNS injury, increased glucose utilization by the injured brain may result in a need for glucose at a threshold higher than the current level of 2.2 mmol/L for managing hypoglycaemia in severe malaria, to prevent cognitive impairment.^42,43^ The findings of this study support further evaluation of whether prevention or early treatment of AKI or the use of a higher threshold for hypoglycemia^44^ may decrease brain injury and prevent long-term cognitive impairment in children with cerebral malaria or severe malarial anaemia.

Among the limitations of this study, we cannot rule out extracerebral production of UCH-L1 and NF-L in an acute inflammatory illness like severe malaria. Other confounding factors relevant to paediatric brain injury include the impact of age on circulating biomarker levels. As few studies have reported paediatric UCH-L1, NF-L, and GFAP levels and none in malaria-endemic countries, the findings of this study need to be validated in larger, multi-site paediatric studies to establish reference ranges for these biomarkers. However, the measurement of basal levels in a well-defined control population and adjustment for age mitigates concerns about non-specific elevations in the disease groups and age-related variability in biomarker levels. This study included a single admission time-point for blood sampling, collected after severely ill children were hospitalized and their condition stabilized, limiting the ability to comment on persisting elevations of these biomarkers or rule out GFAP as a relevant marker despite finding no elevation in children with severe malaria. Using the same assay for biomarker testing, a study of neonatal encephalopathy with serial sampling found that UCH-L1 levels peak between 0 and 6 h and rapidly decrease by 12 and 24 h whereas GFAP levels peak at 12, 48, and 96 h.^45^ Another study found that NF-L may be a useful marker for chronic traumatic brain injury detectable up to 180 days.^46^ Thus, the design of future prospective severe malaria studies should factor in multiple sampling time points during hospitalization and post-discharge. A recent prospective observational study was able to measure brain biomarkers pre- and post-operatively in neonates needing open-heart surgery and found GFAP and tau levels to peak at postoperative Day 2, and NF-L levels increased throughout the study period.^47^ Pre-injury biomarker measurements are not feasible in a study like ours where participants are seen after the onset of acute illness, further amplifying the value of a well-defined control population. Finally, brain imaging studies have elucidated key pathogenic pathways underlying severe malaria in adults and children^37^ and should be conducted in parallel with brain injury biomarker measurements in future cohorts when possible.

Malaria continues to be one of the leading causes of death and disability in Africa, disproportionately affecting children. While diagnostic and triage methods for various types of malaria (e.g. cerebral malaria, severe malarial anaemia) have evolved, there are no objective tools to predict children at highest risk of long-term neurologic deficits or cognitive impairment, which hinders providing timely and effective treatments or rehabilitation interventions for at-risk children. The current longitudinal study takes an important step towards reducing long-term complications due to severe malaria by analysing multimodal associations between brain injury biomarkers (UCH-L1, NF-L, and GFAP) detectable in systemic circulation, and clinical outcomes, such as mortality and cognitive impairment. Admission levels of UCH-L1 have proven utility as a biomarker of BBB dysfunction, neurologic deficits at 24 months, memory impairment over time in cerebral malaria, and attention deficits over time in severe malarial anaemia. NF-L showed equally valuable albeit different utility compared with UCH-L1 as a prognostic marker of death, neurological deficits at discharge, and attention impairment over time in cerebral malaria. Pending confirmation in larger, multi-site studies, these results have important implications for the clinical management of children with severe malaria. First, high levels of NF-L at admission may help prioritize interventions for children with cerebral malaria at the highest risk of death. Second, UCH-L1 and NF-L can serve as a compass to navigate who may benefit from cognitive rehabilitation, such as cognitive exercises, environmental enrichment, nutritional supplementation, physical therapy, and speech therapy.^48^ While some of these preventive approaches require significant resources, it is a logical step to begin employing objective CNS biomarkers, which can be assessed using a semi-automated bench-top device, to guide the management of children with severe malaria.

## Supplementary Material

fcad323_Supplementary_Data

## Data Availability

Deidentified data used in the preparation of the figures and tables will be made available by request of a qualified investigator.
