# Supplementary material for: Blood biomarkers of neuronal injury in paediatric cerebral malaria and severe malarial anaemia
Source: Brain Commun. 2023 Nov 27;5(6):fcad323. doi: 10.1093/braincomms/fcad323 (PMC10710298; doi:10.1093/braincomms/fcad323)
Supplement: fcad323_Supplementary_Data [file fcad323_supplementary_data.docx]

**Supplementary Table 1. Admission UCH-L1 and NF-L levels by A.** Age group; and **B.** Sex

**A**

|  | **N** | **<5 years of age** | **N** | **≥5 years of age** | **P value** |
| --- | --- | --- | --- | --- | --- |
| **UCH-L1** | | | | | |
| CM | 138 | 77.10 (43.52, 182.31) | 44 | 52.93 (28.90, 107.21) | **0.03** |
| SMA | 128 | 48.67 (25.49, 102.75) | 30 | 29.94 (19.55, 47.40) | **0.02** |
| CC | 98 | 12.99 (7.80, 22.69) | 20 | 9.67 (5.31, 16.13) | **0.05** |
| **NF-L** | | | | | |
| CM | 137 | 6.65 (4.10, 15.70) | 44 | 4.60 (3.15, 11.07) | **0.02** |
| SMA | 128 | 6.12 (4.31, 10.94) | 30 | 4.75 (3.59, 10.33) | 0.10 |
| CC | 98 | 4.08 (3.23, 5.07) | 20 | 2.94 (2.51, 3.62) | **<0.001** |
| **GFAP** | | | | | |
| CM | 138 | 121.19 (67.24, 215.99) | 44 | 86.81 (57.69, 115.33) | **0.02** |
| SMA | 128 | 80.78 (62.57, 113.14) | 30 | 69. 61 (54.28, 110.76) | 0.31 |
| CC | 98 | 125.04 (101.85, 212.91) | 20 | 100.55 (79.88, 122.19) | **0.004** |

**B**

|  | **N** | **Female** | **N** | **Male** | **P value** |
| --- | --- | --- | --- | --- | --- |
| **UCH-L1** | | | | | |
| CM | 74 | 90.56 (39.08, 241.72) | 108 | 66.64 (40.09, 109.45) | 0.19 |
| SMA | 58 | 43.05 (29.65, 121.06) | 100 | 45.16 (21.93, 85.05) | 0.14 |
| CC | 61 | 11.20 (9.28, 22.03) | 57 | 15.25 (9.27, 22.02) | 0.16 |
| **NF-L** | | | | | |
| CM | 73 | 6.07 (3.74, 14.09) | 108 | 6.31 (3.91, 13.92) | 0.86 |
| SMA | 58 | 6.93 (4.45, 11.16) | 100 | 5.65 (3.86, 8.82) | 0.09 |
| CC | 61 | 4.01 (2.78, 4.96) | 57 | 3.74 (3.12, 4.96) | 0.94 |
| **GFAP** | | | | | |
| CM | 74 | 108.97 (63.63, 194.57) | 108 | 105.67 (63.57, 208.91) | 0.98 |
| SMA | 58 | 80.78 (70.05, 109.52) | 100 | 76.66 (54.48, 114.54) | 0.21 |
| CC | 61 | 123.20 (100.96, 183.38) | 57 | 118.38 (96.70, 198.19) | 0.76 |

Data presented as median (interquartile range). P values derived from Wilcoxon rank-sum test. Significant P values indicated in bold. CM = cerebral malaria; SMA = severe malarial anemia; CC = community children; UCH-L1 = Ubiquitin C-terminal hydrolase-L1; NF-L = Neurofilament-light chain.

**Supplementary Table 2. Admission UCH-L1 and NF-L levels associated with cognitive impairment over 24 months follow-up.**

|  | **Cerebral malaria** | | | **Severe malarial anemia** | | |
| --- | --- | --- | --- | --- | --- | --- |
|  | n (obs), N | aβ coef (95% CI) | P value | n (obs), N | aβ coef (95% CI) | P value |
| **UCH-L1** | | | | | | |
| **Age < 5 years at severe malaria episode** | | | | | | |
| Overall cognitive ability | 359, 121 | -0.38 (-1.10, 0.35 | 0.30 | 382, 125 | 0.12 (-0.57, 0.81) | 0.73 |
| Attention | 366, 121 | 0.01 (-0.36, 0.38) | 0.95 | 408, 125 | -0.42 (-0.76, -0.07) | **0.02** |
| Associative memory | 368, 121 | -0.05 (-0.24, 0.13) | 0.57 | 394, 123 | -0.07 (-0.33, 0.18) | 0.58 |
| **Age <5 years at severe malaria episode, ≥ 5 years at testing** | | | | | | |
| Overall cognitive ability | 92, 65 | -0.60 (-1.36, 0.15) | 0.12 | 64, 41 | -0.57 (-1.34, 0.19) | 0.14 |
| Attention | 93, 65 | -0.64 (-1.33, 0.06) | 0.07 | 65, 42 | -0.57 (-1.29, 0.15) | 012 |
| Working memory | 93, 65 | -1.13 (-2.05, -0.21) | **0.02** |  |  |  |
| **Age ≥ 5 years at severe malaria episode** | | | | | | |
| Overall cognitive ability | 163, 42 | -0.17 (-1.70, 1.35) | 0.82 | 117, 31 | 0.07 (-1.42, 1.57) | 0.92 |
| Attention | 161, 42 | 0.003 (-0.87, 0.88) | 0.99 | 118, 31 | 0.51 (-0.65, 1.66) | 0.38 |
| Working memory | 164, 42 | -0.18 (-1.14, 0.77) | 0.70 | 120, 31 | -0.04 (-1.39, 1.31) | 0.95 |
| **NF-L** | | | | | | |
| **Age < 5 years at severe malaria episode** | | | | | | |
| Overall cognitive ability | 359, 121 | -0.24 (-0.95, 0.47) | 0.50 | 382, 125 | -0.79 (-1.56, -0.02) | 0.05 |
| Attention | 366, 121 | 0.19 (-0.16, 0.54) | 0.29 | 408, 125 | -0.21 (-0.60, 0.18) | 0.29 |
| Associative memory | 368, 121 | -0.02 (-0.20, 0.16) | 0.82 | 394, 123 | -0.04 (-0.33, 0.24) | 0.76 |
| **Age <5 years at severe malaria episode, ≥ 5 years at testing** | | | | | | |
| Overall cognitive ability |  |  |  | 64, 41 | -0.29 (-1.38, 0.80) | 0.59 |
| Attention | 93, 65 | -0.54 (-1.28, 0.20) | 0.15 | 65, 42 | -0.76 (-1.75, 0.23) | 0.13 |
| Working memory | 93, 65 | -0.44 (-1.43, 0.54) | 0.37 |  |  |  |
| **Age ≥ 5 years at severe malaria episode** | | | | | | |
| Overall cognitive ability | 163, 42 | -0.88 (-2.66, 0.89) | 0.32 | 117, 31 | -0.53 (-2.39, 1.32) | 0.56 |
| Attention | 161, 42 | -1.08 (-2.05, -1.05) | **0.03** | 118, 31 | -0.77 (-2.18, 0.65) | 0.28 |
| Working memory | 164, 42 | -0.27 (-1.39, 0.86) | 0.63 | 120, 31 | 1.01 (-2.64, 0.62) | 0.22 |

Table presents the adjusted β coefficients (aβ coef) and 95% confidence intervals (95% CI) for biomarker concentrations and longitudinal changes in cognitive z-scores at 1 week, 6-, 12-, and 24 months follow-up where within-subject observations (n) were correlated using a subject-specific intercept. The estimates were obtained using linear mixed effects (LME) models adjusted for age and sex. Significant P values indicated in bold. UCH-L1 = Ubiquitin C-terminal hydrolase-L1; NF-L = Neurofilament-light chain.

**Supplementary Table 3. Clinical laboratory factors associated with admission UCH-L1 and NF-L levels**

|  | **Cerebral malaria** | | **Severe malarial anemia** | |
| --- | --- | --- | --- | --- |
|  | **aβ coef (95% CI) (n=182)** | **P value** | **aβ coef (95% CI) (n=158)** | **P value** |
| **UCH-L1** | | | | |
| Glucose (mmol/L) | -0.32 (-0.62, -0.03) | **0.03** | -0.44 (-0.76, -0.13) | **0.006** |
| Lactate (mmol/L) | 0.28 (0.07, 0.49) (167) | **0.008** | 0.31 (0.05, 0.57) (146) | **0.02** |
| Sodium (mmol/L) | -0.01 (-0.03, -0.01) | 0.05 | -0.13 (-0.03, 0.005) | 0.17 |
| Blood urea nitrogen (mg/dL) | 0.66 (0.42, 0.91) (179) | **<0.001** | 0.49 (0.23, 0.76) | **<0.001** |
| Acute kidney injury | 0.32 (0.18, 0.45) (173) | **<0.001** | 0.40 (0.23, 0.57) (153) | **<0.001** |
| Hemoglobin (g/dL) | -0.38 (-0.88, 0.13) | 0.14 | -0.08 (-0.71, 0.53) | 0.78 |
| Platelet count | -0.25 (-0.42, -0.07) | **0.007** | -0.37 (-0.58, -0.16) | **0.001** |
| Lactate dehydrogenase (U/L) | 0.96 (0.69, 1.22) (172) | **<0.001** | 0.78 (0.42, 1.14) (151) | **<0.001** |
| **NF-light** | | | | |
| Glucose (mmol/L) | -0.20 (-0.49, 0.08) | 0.16 | -0.03 (-0.32, 0.27) | 0.85 |
| Lactate (mmol/L) | -0.18 (-0.38, 0.03) (167) | 0.09 | -0.01 (-0.22, 0.22) | 0.99 |
| Sodium (mmol/L) | 0.01 (-0.001, 0.02) | 0.08 | 0.001 (-0.01, 0.02) | 0.92 |
| Blood urea nitrogen (mg/dL) | 0.33 (0.08, 0.57) (179) | **0.009** | 0.17 (-0.06, 0.41) | 0.14 |
| Acute kidney injury | 0.22 (0.09, 0.35) (174) | **0.001** | 0.19 (0.04, 0.34) | **0.005** |
| Hemoglobin (g/dL) | -0.33 (-0.80, 0.13) | 0.16 | -0.09 (-0.61, 0.43) | 0.73 |
| Platelet count | -0.02 (-0.18, 0.14) | 0.78 | -0.11 (-0.30, 0.07) | 0.24 |
| Lactate dehydrogenase (U/L) | 0.41 (0.13, 0.68) (173) | **0.004** | 0.006 (-0.31, 0.32) | 0.97 |

Adjusted β coefficients (aβ coef), 95% confidence intervals (95% CI), and number of samples tested per group are shown. P values indicate univariable linear regression analysis adjusted for age and sex. Significant P values indicated in bold after correction for 8 laboratory factors within each marker of interest for each malaria group using the Benjamini-Hochberg procedure. All continuous variables were log_10_ transformed. UCH-L1 = Ubiquitin C-terminal hydrolase-L1; NF-L = Neurofilament-light chain.
